# Does treatment with antiviral medication affect mortality among immunocompetent individuals with cytomegalovirus reactivation? A systematic review and meta-analysis

**DOI:** 10.1186/s12879-025-12205-6

**Published:** 2025-11-27

**Authors:** Alice Ying-Jung Wu, Ming-Chieh Tsai, Sam Newton

**Affiliations:** 1 Division of Infectious Diseases, Department of Internal Medicine, Mackay Memorial Hospital, No. 45, Minsheng Rd., Tamsui District, New Taipei City, 25160 Taiwan; 2 Department of Medicine, Mackay Medical University, New Taipei City, Taiwan; 3 Division of Endocrinology and Metabolism, Department of Internal Medicine, Mackay Memorial Hospital, Taipei, Taiwan; 4 Institute of Epidemiology and Preventive Medicine, College of Public Health, National Taiwan University, Taipei City, Taiwan; 5 School of Public Health, Kwame Nkrumah University of Science and Technology, Kumasi, Ghana

**Keywords:** Cytomegalovirus, Reactivation, Antiviral, Ganciclovir, Valganciclovir, Valacyclovir, Systematic review, Mortality

## Abstract

**Background:**

Latent cytomegalovirus (CMV) can reactivate in times of immunosuppression or during critical illness and is associated with worse prognosis, but the benefit of using antiviral medications in otherwise immunocompetent individuals during CMV reactivation is unclear. This study aims to systematically review the effect of using antiviral medications on clinical outcomes in immunocompetent population.

**Methods:**

Database searches were performed in EMBASE, Medline, Scopus, CINAHL Complete, as well as in two trial registries ClinicalTrials.gov and ICTRP Search Portal. Search terms focused on CMV and antiviral medications among non-immunocompromised hosts admitted to the hospital. Effect estimates were combined using a Random Effects meta-analysis.

**Results:**

Four randomized controlled trials and two cohort studies were included (total *N* = 785). There was no significant difference in the use of antiviral medications on composite measure of mortality (*p* = 0.18) either when given as preemptive treatment or as prophylaxis (*p* = 0.42), or when subgrouped by randomized studies versus observational studies were compared (*p* = 0.86). There was no difference in overall intensive care unit length of stay (*p* = 0.40) or duration of hospitalization (*p* = 0.25).

**Conclusion:**

Using antiviral medications in immunocompetent individuals with CMV reactivation did not affect mortality, ICU duration, or hospital duration. No conclusion can be reached regarding decrease in respiratory support post antiviral use.

**Clinical trial:**

This study is not a clinical trial.

**Supplementary Information:**

The online version contains supplementary material available at 10.1186/s12879-025-12205-6.

## Introduction

### Background of cytomegalovirus and epidemiology

Infection with CMV is widespread [1]. Evidence of having been infected with cytomegalovirus increases with age. Seroprevalence among 18–19-year-olds ranges from 13.7% to 49.0%, while it is 22.6% to 61.5% among 38–39-year-olds in the United States and Canada [2]. It is estimated that CMV has a worldwide seroprevalence of 83% in the general population [1]. Post acute infection, CMV establishes latent infection by harboring in monocytes and macrophages [3]. In the setting of immunosuppression, such as during HIV infection, post chemotherapy for malignancy, post glucocorticoid use, or in those critically ill, latent CMV may become reactivated [4–7]. In critically ill patients, prevalence of reactivation ranges from 13.75% to 36% [7–10]. Reactivation of CMV viremia is associated with worse clinical outcomes, including increased mortality [8, 11–14], prolonged duration of mechanical ventilation [4] and longer lengths of intensive care unit stay [15], longer lengths of hospital stay [13], and increased secondary infections [16].

The mechanisms by which CMV (CMV) worsens clinical outcomes remain unclear. CMV may directly affect tissues, particularly the lungs, as several studies have linked CMV reactivation to lung injury and prolonged mechanical ventilation [11–13]. It may also dysregulate the immune response, causing increased levels of IL-6, IL-8, IL-10, CD8^+^ T cells, TNF alpha, gamma-delta T cells and macrophages [17], leading to tissue inflammation and higher mortality. Alternatively, CMV can promote immunosuppression by elevating IL-10 or altering human leukocyte antigen and memory T cell expression, reducing host defenses and facilitating secondary bacterial or fungal infections [17]. This in turn lowers host defenses, facilitating the development of secondary bacterial or fungal infection [17, 18]. Some researchers suggest that CMV is not directly pathogenic but serves as a marker of disease severity [19].

In immunocompromised patients, such as those who have received solid organ or hematopoietic stem cell transplantation (HSCT), preemptive CMV treatment is standard. No consensus exists for immunocompetent patients. Expert commentary and reviews have debated whether CMV is a bystander or a pathogen in critical illness, and guidance remains inconsistent [20, 21]. Although CMV presence is linked to worse outcomes, it remains unclear whether antiviral therapy improves outcomes, particularly given potential side effects such as myelosuppression and nephrotoxicity.

Several clinical strategies exist for managing CMV infection. In a prophylactic approach, all patients are treated up front, regardless of disease or reactivation, which increases medication-related side effects, costs, and the risk of antiviral resistance. In a preemptive strategy, patients are treated if CMV infection is detected without definitive disease, most commonly indicated by viral detection in blood or tracheobronchial specimens. No established criteria exist for screening frequency or antiviral initiation thresholds in nonimmunocompromised critically ill patients.

The gold standard for CMV disease is pathologic evidence of intracellular CMV invasion, demonstrated by the classic “owl’s eye” nuclear inclusion. As this criterion involves an invasive procedure and is not undertaken by most patients due to high risk, antivirals are often administered prior to the establishment of definitive CMV disease.

This present study reviews the current evidence regarding whether treatment with antivirals affects clinical outcomes in patients among immunocompetent individuals with CMV reactivation.

### Aims and objectives

This project aims to determine whether antiviral treatment for CMV reactivation in immunocompetent patients improves clinical outcomes, including mortality, healthcare-associated infections, ICU and hospital length of stay, and the need for respiratory support.

## Materials and methods

### Search strategy and study selection

A systematic search was conducted in the electronic databases Ovid MEDLINE, Ovid EMBASE, SCOPUS, and CINAHL Plus and updated to August 22, 2025. The EMBASE search engine was used to search separately for both MEDLINE records and EMBASE records to keep exported citations compatible. Citation titles were searched “Cytomegalovirus” or “CMV”, NOT “transplant” or “transplantation” or “hematopoietic” or “solid organ” or “HSCT” or “HCT” or “HIV” or “lymphoma” or “leukemia” or “PJP” or “Pneumocystis” or “malignancy” or “AIDS” or “lupus” or “colitis” or “child” or “children” or “fetus” or “fetal” or “congenital” or “neonates” or “SLE” or “retinitis” or “cirrhosis” or “cancer” or “case” or “review”, AND “ICU” or “intensive care” or “critical” or “critically” or “preemptive” or “prophylaxis” or “reactivation” or “ganciclovir” or “valganciclovir” or “valaciclovir” or “valacyclovir” or “gancyclovir” or “valgancyclovir” or “letermovir” or “maribavir” or “foscarnet” or “cidofovir” or “antiviral” or “treatment”. The searches were limited to English language and adults.

In addition, a search of grey literature to look for ongoing clinical trials with preliminary research results was conducted. The database ClinicalTrials.gov was searched using the search term “cytomegalovirus” and “ganciclovir”. ICTRP Search Portal was searched for grey literature using the search term “cytomegalovirus”. Appendix A provides a detailed description of the search process for each database.

### Eligibility criteria and considering studies for this review

This systematic review followed the PRISMA (Preferred Reporting Items for Systematic Reviews and Meta-Analyses) guidelines for methodology and reporting [22].

Two researchers independently assessed articles for eligibility. After screening, results were compared, and articles were included only if both researchers reached unanimous agreement. Articles excluded if the subject were children, immunocompromised (solid organ or bone marrow transplant patients, those with advanced HIV/AIDS, liver cirrhosis, or those receiving cytotoxic or immunosuppressive therapies); or had evidence of documented CMV tissue invasive disease. Case reports and case series were excluded, as these studies did not report measures of effect and were considered inadequate to report primary or secondary outcome measures.

### Data extraction

After determining eligible articles, the data were extracted using a spreadsheet in Excel. The data extracted were: author name, study year, country of study, reference, patient population, number of patients, gender of patients, patient ages with age distribution, major inclusion criteria, major exclusion criteria, intervention, control, mortality (as measured in any way), the individual study’s primary outcome measure, and any secondary clinical outcome measures as reported by the studies, such as lengths of ICU stay, duration of mechanical ventilation, lengths of hospital stay, ventilator-free days (VFDs), and respiratory support-free days (RSFDs).

Duration of mechanical ventilation referred to number of days a patient required mechanical ventilation. VFDs referred to the number of days a patient was alive and free from invasive mechanical ventilation at a certain timepoint. Only the Stapleton abstract used RSFDs, and the definition was not defined in the abstract itself, but prior literature defined this measure as the number of days within a fixed time window when the patient did not require any form of respiratory support, including high-flow nasal oxygen, noninvasive ventilation, or invasive mechanical ventilation [23]. We used the original trial definitions when extracting data and present duration of mechanical ventilation, VFDs and RSFDs using the terminology from the respective trials.

#### Quality assessment

Quality assessment of the included studies were performed Version 2 of the Cochrane risk-of-bias tool for randomized trials (RoB2) and for the Newcastle-Ottawa Assessment tool for cohort studies.

### Data synthesis

Crude numbers of deaths and survivors from each study were included in the meta-analysis. A random-effects meta-analysis estimated the odds of mortality, however defined, in patients receiving any antiviral versus no treatment. For studies with multiple treatment groups, results from groups receiving any antiviral against CMV were combined. When deaths were reported at multiple time points, the 28-day mortality or the closest available measure was used. Only crude odds ratios were compared, as only one observational study reported adjusted hazard ratios. Subgroup analyses were performed for randomized controlled trials (RCTs) versus observational studies and for prophylactic versus preemptive antiviral strategies. Publication bias was assessed using a funnel plot.

For continuous variables—ICU length of stay and hospital length of stay, means and standard deviations were estimated from medians and interquartile ranges using the method of Wan et al. [24]. This method assumes approximately symmetric distributions; where sample sizes are small or distributions markedly skewed, the approximation may be less accurate. We therefore regard pooled estimates that rely on converted mean/SD values as approximate and interpret them cautiously. Subgroup analyses were not performed due to limited data. Heterogeneity among studies was assessed using the I² statistic.

All statistical analyses and graphics were carried out using STATA, version 16 (StataCorp LLC, College Station, TX, 2019). Meta-analyses were performed with the meta esize and eform(Odds ratio) commands, publication bias was assessed with meta funnelplot. This review was registered with PROSPERO with the number CRD42025637664.

## Results

### Summary of included studies

**Fig. 1:**
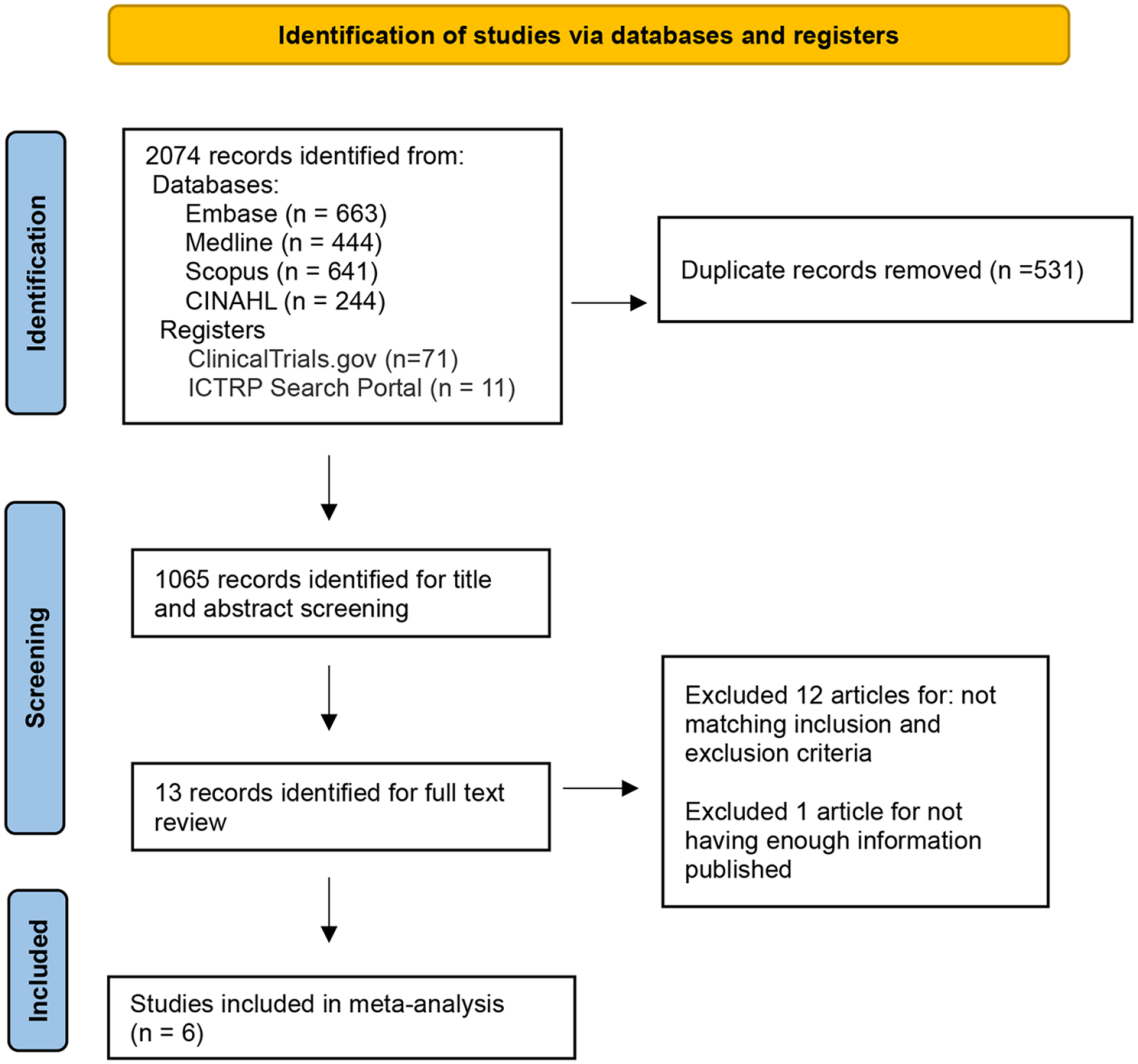
PRISMA flow diagram for study selection

A total of 2074 citations were found by the initial search. After screening for duplicates, and excluding those that were not eligible for the study, six studies were included in the systematic review. The PRISMA flow diagram is presented in Fig. 1.

Six articles were found after extensive search, as listed in Table 1, comprising of four RCTs and two cohort studies. Overall, 785 subjects were studied. The mean age was 61.4, of which 464 were men (59.1%).

Four RCTs were found after extensive search.

The Cowley study [25] was a single-center, open-label, randomized controlled trial comparing two anti-CMV treatments with a control group in mechanically ventilated ICU patients without immunosuppression. Patients who were seropositive for CMV received prophylactic antiviral therapy: valganciclovir 450 mg once daily enterally or intravenous ganciclovir 2.5 mg/kg daily, and valacyclovir 2 g four times daily enterally or intravenous acyclovir 10 mg/kg three times daily.

The primary outcome was time to first CMV reactivation in blood; secondary outcomes included fifteen measures, including 28-day mortality. Participants were randomized via computer-generated allocation and stratified by age. Patients and physicians were not blinded, but the primary endpoint and laboratory staff were blinded. Of 473 screened patients, 124 were randomized. The valacyclovir arm was discontinued early for safety concerns, so the valganciclovir arm was subsequently compared with the control group.

The study suggests that prophylaxis with valacyclovir or low-dose valganciclovir suppresses CMV reactivation in non-immunocompromised critically ill patients, though the valacyclovir arm may be associated with higher mortality.

The Limaye study [26] was a double-blind, placebo-controlled, randomized trial comparing CMV prophylaxis with placebo in non-immunocompromised adults hospitalized with respiratory failure and severe sepsis who were intubated and CMV IgG positive. Patients received ganciclovir or ganciclovir followed by valganciclovir, while the control group received placebo. Ganciclovir was dosed at 5 mg/kg intravenously every 12 h for 5 days, then 5 mg/kg daily, adjusted for renal function; patients could switch to 900 mg oral valganciclovir until day 14 or hospital discharge (maximum 28 days).

Randomization was 1:1 and blinded. The primary outcome was plasma IL-6 levels; secondary outcomes included VFDs and mortality. Of 160 patients randomized, 84/84 in the treatment group and 72/76 in the placebo group were included in the primary analysis. At day 180, 16/84 and 8/76 were lost to follow-up, respectively. The study found that ganciclovir did not reduce IL-6 levels but significantly lowered CMV reactivation in plasma.

The Papazian study [27] was a double-blind, placebo-controlled, randomized trial evaluating a preemptive antiviral strategy in adults mechanically ventilated for ≥ 96 h with CMV viremia. Patients received either intravenous ganciclovir or placebo. Ganciclovir was administered at 5 mg/kg every 12 h for 5 days, then 5 mg/kg daily, with renal adjustments, for a maximum of 14 days; patients could continue with 900 mg oral valganciclovir until day 14 or discharge.

Randomization was web-based and stratified by study site, ventilation duration, and SOFA score. CMV viremia was defined as ≥ 500 IU/mL CMV DNA in whole blood by PCR. Of 2,809 screened patients, 2,733 were excluded, including 1,783 extubated or deceased before CMV results were available. The primary outcome was VFDs at day 60; outcomes were measured per the prespecified plan. The trial was stopped early as 822 patients would have been needed to detect a difference. Preemptive ganciclovir did not improve outcomes in mechanically ventilated patients with CMV reactivation.

The Park study [28] was a single-center, retrospective cohort study of non-immunocompromised patients with CMV reactivation who were treated preemptively with ganciclovir or not treated. Ganciclovir was administered at 5 mg/kg every 12 h, adjusted for renal function. CMV reactivation was defined as ≥ 270 copies/mL in whole blood by RT-PCR. Patients who died within 30 days were excluded. The median age was 70 years, and less than half were admitted to the ICU.

The study assessed long-term survival at 12 and 30 months. The 30-month survival rate was adjusted for age, ganciclovir treatment, ICU admission, qSOFA score, Charlson Comorbidity Index, and CMV PCR > 50,000 copies/mL. Ganciclovir was not associated with improved long-term survival.

The Schoninger study [29] was a single-center, retrospective cohort study of ICU patients with COVID-19 pneumonia and CMV viremia. CMV viremia was defined as detection of CMV DNA in plasma, serum, or whole blood, regardless of viral load. Patients were either preemptively treated with ganciclovir and/or valganciclovir for at least 5 days or not treated, with dosages unspecified. Since the primary endpoint was all-cause in-hospital mortality, patients still hospitalized at study end were excluded. The study found that treatment of CMV viremia did not reduce in-hospital mortality among ICU patients with COVID-19.

The Stapleton study [30] was a double-blind, placebo-controlled, 16-center randomized trial of immunocompetent CMV-seropositive adults with sepsis-associated respiratory failure. Patients received prophylactic intravenous ganciclovir (5 mg/kg twice daily for 5 days, then once daily) or placebo until day 28 or discharge. The primary outcome, RSFDs by day 28, was not improved by ganciclovir. Secondary outcomes—mortality at day 28 and 180—were higher in the treatment arm (*p* = 0.005 and 0.004, respectively), prompting early termination on DSMB recommendations. CMV reactivation in plasma was significantly lower with ganciclovir compared with placebo (9.1% vs. 52.0%, *p* = 0.001). Of note, the Stapleton study is currently only available as a conference abstract.

**Table 1:** Basic study characteristics and study results

Study, year and country	Type of Study	Population	Intervention	Main Study Endpoint Finding	Mortality	ICU LOS, median (IQR), days	Hosp LOS, median (IQR), days	Changes in Respiratory Support
Cowley 2017, England	RCT	Non-immunocompromised CMV seropositive adults on mechanical ventilation in the ICU, not pregnant or breastfeeding, non neutropenic (ANC > 1000)	Valaciclovir, Valganciclovir, standard care (3 arms)	Antiviral treatment group lowered CMV reactivation (HR 0.14, 95% CI 0.04 to 0.50)	Day 28 mortality higher valacyclovir arm, 14 out of 34 (41.2%)	Comparable. Control: 11.5 (7–16), *n* = 44. Valacyclovir: 12.0 (7–31), *n* = 34. Valganciclovir: 16.0 (11–27), *n* = 46.	Not reported	Not reported
Limaye 2017, US	RCT	Non-immunocompromised CMV seropositive adults on mechanical ventilation in the ICU, with no extreme obesity (BMI > 60), liver cirrhosis Child C or preexisting interstitial lung disease, non neutropenic (ANC > 1000)	Ganciclovir or ganciclovir followed by valganciclovir versus placebo	No difference in change of plasma IL-6 levels between day 0 and 14 (*p* > 0.99)	Day 28 mortality comparable. Ganciclovir: 10 / 84 (12%). Placebo: 11/72 (15%); *p* = 0.54	Comparable. Ganciclovir: 8 (4 to 14), *n* = 84, placebo: 8 (5 to 15), *n* = 72; *p* = 0.76	Comparable. Ganciclovir: 14 (8 to 22), *n* = 84, placebo: 13 (8 to 23), *n* = 72; *p* = 0.92	Mechanical ventilation duration, median (IQR), days: Comparable. Ganciclovir: 5 (3 to 9), *n* = 84, placebo: 6 (3 to 12), *n* = 72; *p* = 0.16. VFDs by day 28, median (IQR), days: Treatment group decreases VFDs (*p* = 0.05). Ganciclovir: 23 (16 to 25), *n* = 84, placebo: 20 (8 to 24), *n* = 72; *p* = 0.05
Papazian 2021, France	RCT	Non-immunocompromised CMV seropositive adults on mechanical ventilation in the ICU, not pregnant or breastfeeding, non-neutropenic (ANC > 1000)	Ganciclovir vs. placebo	No difference in VFDs by day 60. Median (IQR) ganciclovir 10 (0–51), *n* = 39; placebo 0 (0–43) *n* = 37, *p* = 0.459	Day 28 mortality: Treatment 11/39 (28.2%), control: 12/37 (32.4%), *p* = 0.689.	Ganciclovir: 36 (24–51), *n* = 39; placebo: 44.0 (21.0–66.5), *n* = 37; *p* = 0.377	Ganciclovir: 65.0 (28.0–78.0), *n* = 39, placebo: 60.0 (33.0–75.5), *n* = 37; *p* = 0.988	Duration of mechanical ventilation (median, IQR): ganciclovir: 12 (6–29), 20 (7–40); *p* = 0.25. VFDs by day 28, median (IQR), days: Ganciclovir: mean 8.1 SD 10.6, placebo: mean 6.1 SD 9.6, mean difference 0.3 (SD 0.2), *p* = 0.256
Park 2021, South Korea	retrospective cohort	Non-immunocompromised patients with evidence of CMV reactivation, who did not die within 30 days	Ganciclovir vs. not treated	Ganciclovir treatment was not associated with long-term prognosis.12-months survival: ganciclovir: 25/62 (40.3%), non-treated 30/61 (49.2%), *p* = 0.323; 30-months ganciclovir: 14/58 (28%), non-treated 21/54 (38.9%), *p* = 0.240	90-days adjusted hazard ratio of ganciclovir treatment: 2.006 (0.654–6.158), *p* = 0.224	Not reported	Not reported	Not reported
Schoninger 2022, USA	retrospective cohort	Non-immunocompromised adults with COVID-19 pneumonia, admitted to ICU, and evidence of CMA reactivation who was discharged before end of study period	Ganciclovir and/or valganciclovir for at least 5 days vs. not treated	All-cause in-hospital mortality was unchanged after treatment	In hospital mortality for treated: 16/43 (37.2%), control 16/37 (43.2%), *p* = 0.749	Treatment group had increased ICU length of stay. Treatment: 51 (33–79), *n* = 43, control: 38 (22–52), *n* = 37; *p* = 0.014	Comparable. Treatment: 63 (40–88), *n* = 43; control: 49 (34–74), *p* = 37, *p* = 0.121	Mechanical ventilation duration, median (IQR), days: Comparable. Treatment: 45 (27–77), *n* = 43; control: 37 (18–59), *n* = 37; *p* = 0.176.
Stapleton 2025, USA	RCT	Non-immunocompromised patients with evidence of CMV reactivation with sepsis-associated respiratory failure	Ganciclovir vs. placebo	Respiratory support-free days by day 28 was unchanged	Day 28 mortality higher for ganciclovir: 33/106 (31.1%), control: 13 / 99 (13.1%), *p* = 0.005; Day 180 mortality higher for ganciclovir: 42/106 (39.6%), control 20/99 (20.2%), *p* = 0.004	Not reported	Not reported	No difference in respiratory support-free days by day 28: ganciclovir: mean 12.4 SD 11.1, *n* = 106, placebo mean 14.9 SD 10.3, *n* = 99; *p* = 0.11

Abbreviations: RCT = randomized controlled trial, HR = hazard ratio, IQR = interquartile range, LOS: length of stay, SD = standard deviation

### Analysis of pooled results

#### Analysis of mortality

**Fig. 2:**
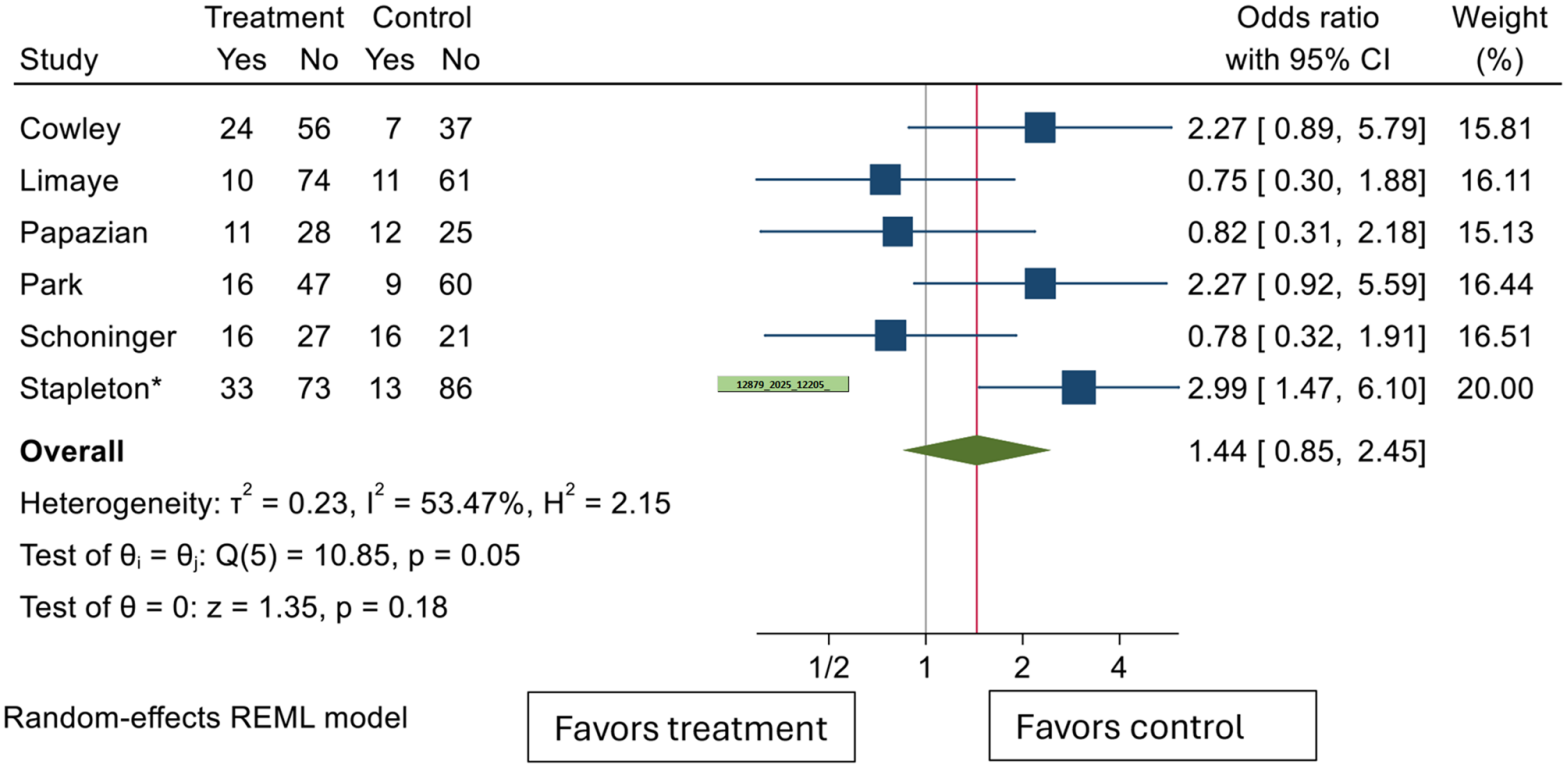
Odds ratio of composite mortality after using antiviral treatment. CI: confidence interval. *Stapleton et al. (abstract, 2025) — data from conference abstract; full manuscript not yet published

A composite mortality measure was determined comparing use of any type of antiviral medications versus placebo or not using any antiviral medication and compared in a random effects model. Overall, there appeared to be no mortality benefit in using antiviral medication in this group of patients (Fig. 2). Odds ratio of overall mortality among patients who received any type of medication was 1.44 (95% CI 0.85–2.45, *p* = 0.18). Between study heterogeneity was substantial (I^2^test 53.47%, Cochrane’s Q *p* = 0.05).

**Fig. 3:**
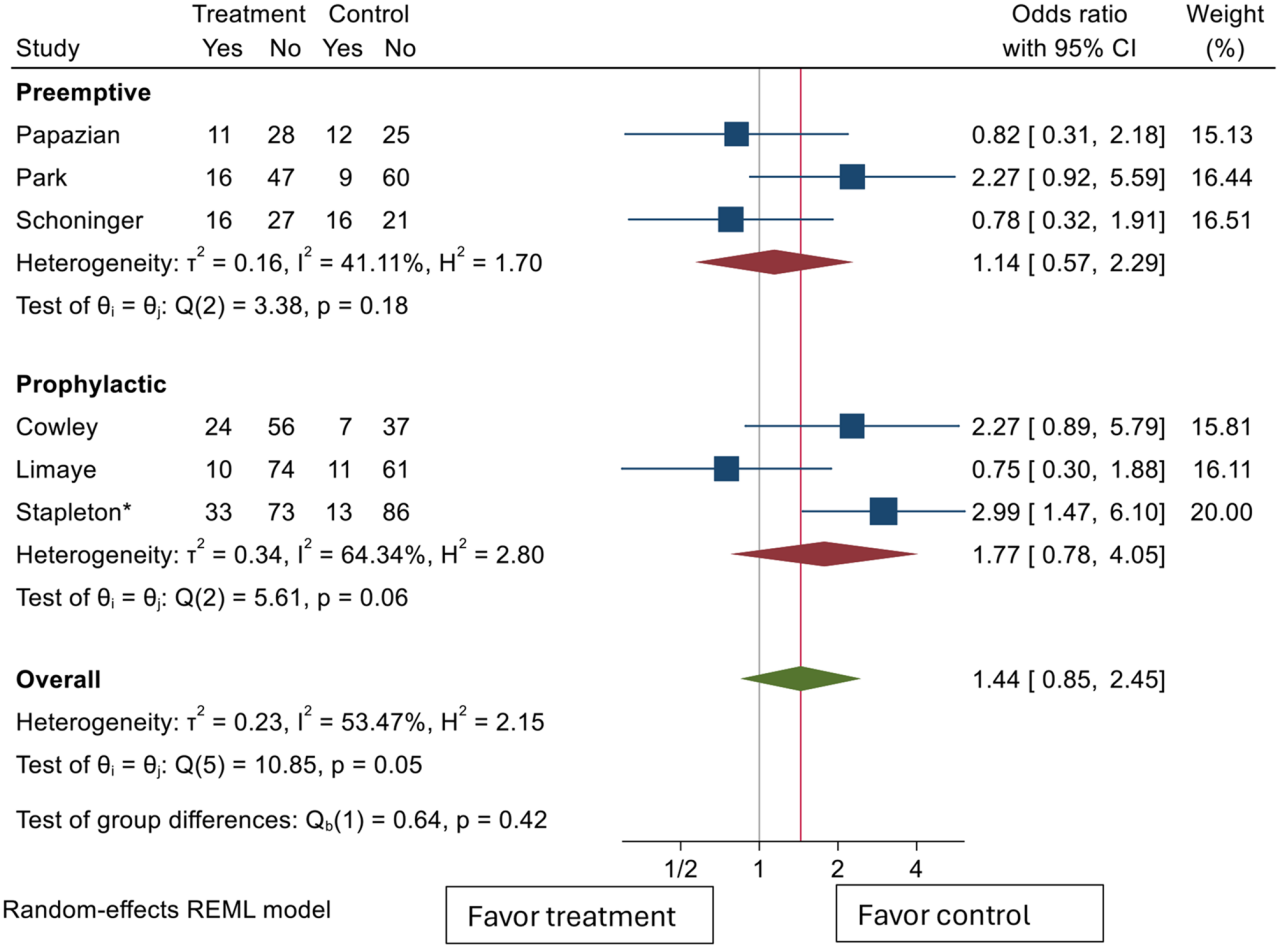
Pooled effect estimates of the association between any type of antiviral medication use and mortality by treatment strategy. CI: confidence interval. *Stapleton et al. (abstract, 2025) — data from conference abstract; full manuscript not yet published

**Fig. 4:**
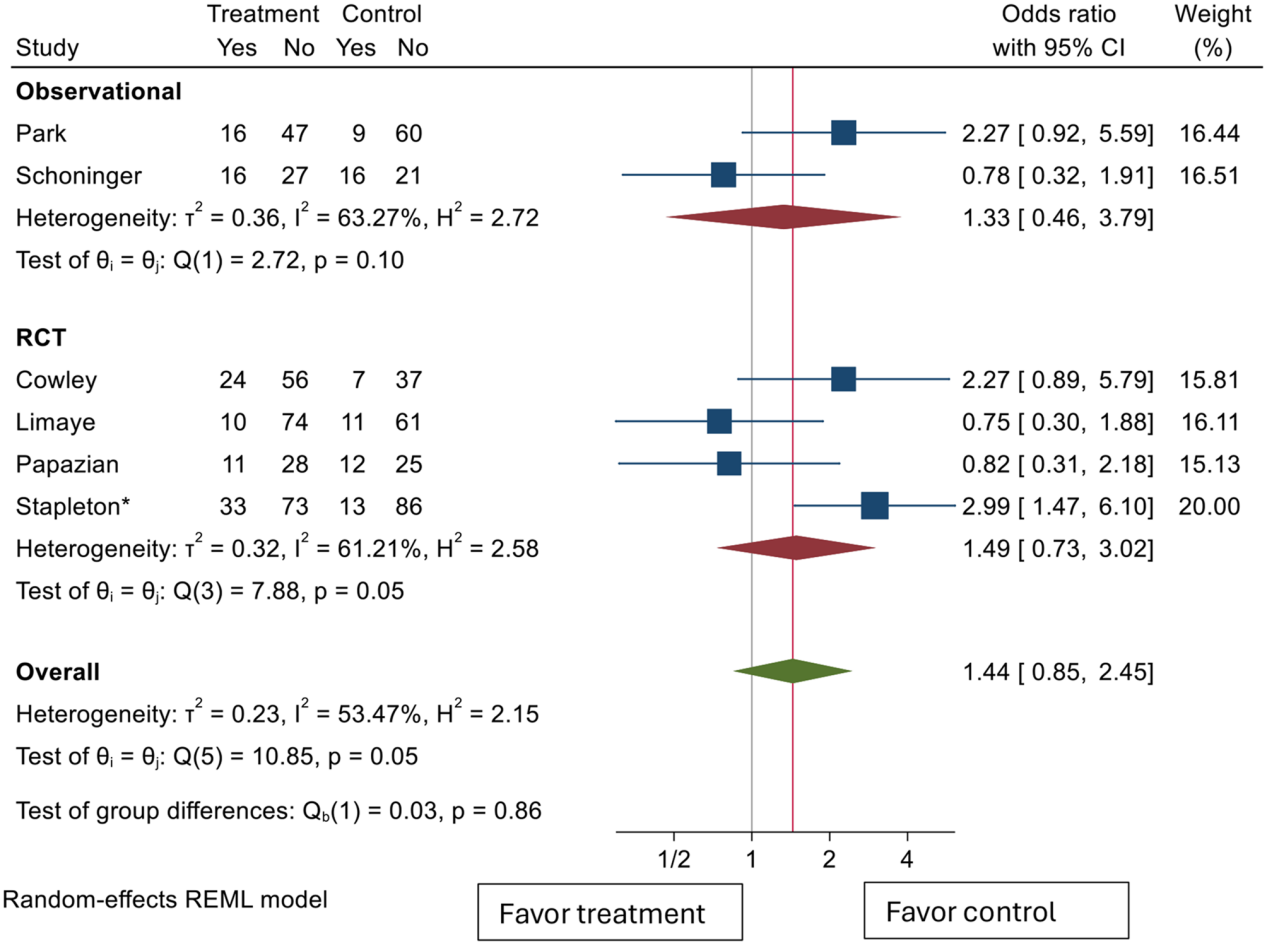
Pooled effect estimates of the association between any type of antiviral medication use and mortality by publication type. CI: confidence interval. *Stapleton et al. (abstract, 2025) — data from conference abstract; full manuscript not yet published

As our studies were comprised of different antiviral usage strategies, subgroup analysis comparing studies that used a prophylactic strategy (Cowley [25], Limaye [26]), Stapleton [30] vs. ones that used preemptive strategy (Papazian [27], Park [28], Schoninger [29]) was performed (Fig. 3). Both preemptive studies and prophylactic studies showed nonsignificant odds ratios with regards to mortality benefit post antiviral use, with odds ratios of 1.14 (95% CI 0.57–2.29) and 1.77 (95% CI 0.78–4.05), respectively. In general, there was more heterogeneity in the prophylactic group as I2 value of 63.34% was greater than that of the preemptive studies (41.11%). To explore issues of heterogeneity, studies were grouped again by type of study (Fig. 4). When grouped by type of studies performed, both observational studies and RCTs again showed nonsignificant odds ratios; with odds ratios of 1.33 (95% CI 0.46–3.79) and 1.49 (95% CI 0.73–3.02) in observational and RCTs, respectively. Both groups showed high heterogeneity with I squared statistics of 63.27% and 61.21%, respectively. Since all the RCTs used 28-day mortality as their outcome measure, this subgroup analysis also showed no difference in 28-day mortality across the four included studies. A summary of the studies, study type, antiviral strategy, setting, and mortality outcome measured is summarized in Table 2.

**Fig. 5:**
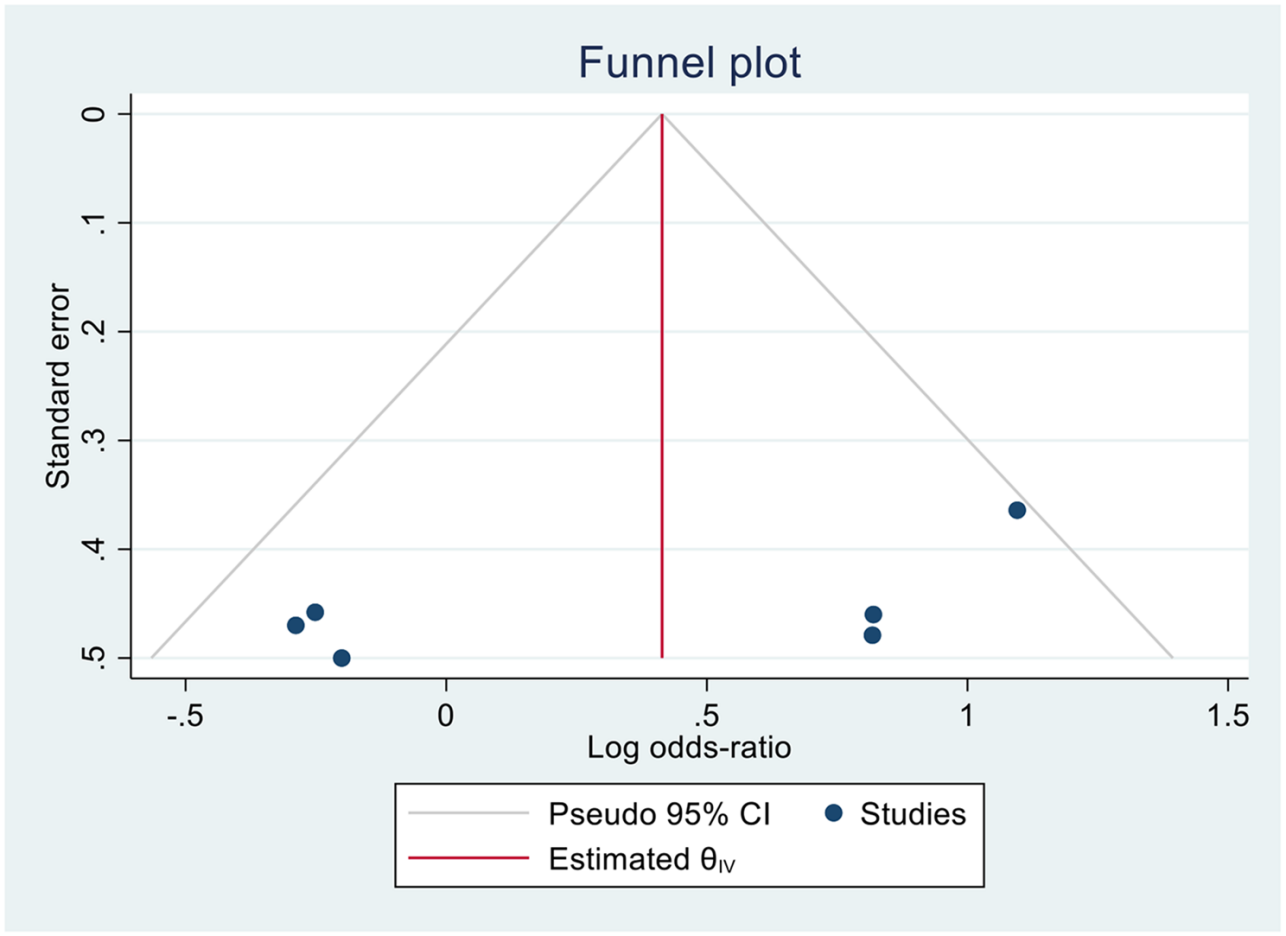
The funnel plot of the overall mortality shows that there was overall no evidence of publication bias by visual inspection

As fewer than ten studies were included, no formal test of publication bias was carried out. Visual inspection of the funnel plot did not suggest strong asymmetry, although interpretation is limited due to the small number of studies (Fig. 5).

The mortality rates and designs of the studies followed by the Forest Plot are as follows.

**Table 2:** Summary of studies, study types, treatment strategies, setting, and mortality measure

Trial	Type of Study	Strategy Employed	Setting	Mortality Measured
Cowley2017	RCT	Prophylactic	ICU	Day 28 mortality
Limaye 2017	RCT	Prophylactic	ICU	Day 28 mortality
Papazian 2021	RCT	Preemptive	ICU	Day 28 mortality
Park 2021	Observational	Preemptive	Hospitalized	90 days mortality
Schoninger 2022	Observational	Preemptive	ICU	In-hospital mortality
Stapleton 2025	RCT	Prophylactic	ICU	Day 28 mortality

#### Analysis of mean differences in ICU lengths of stay, days of hospital duration, and alteration in respiratory support

**Fig. 6:**
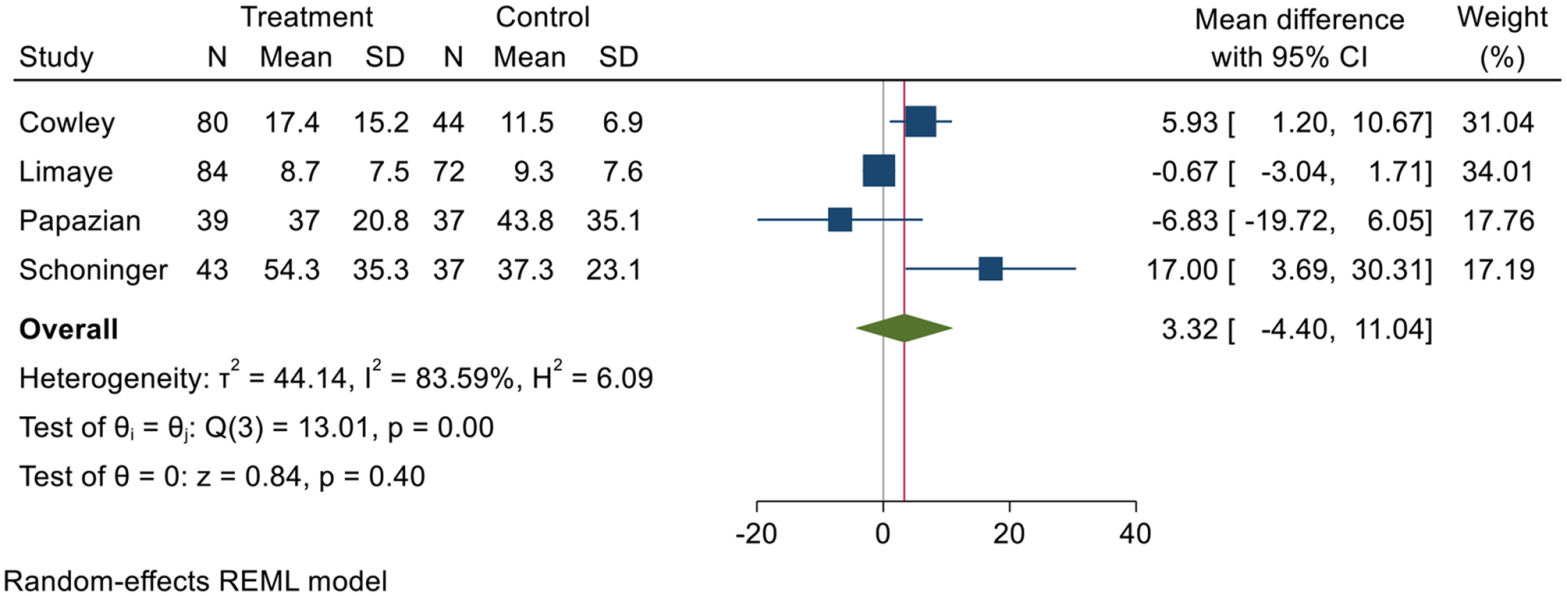
Comparison of mean differences in ICU lengths of stay (in days). SD = standard deviation, CI = confidence interval

Overall, there was no difference in intensive care unit length of stay, with mean difference of 3.32 days (*p* = 0.40) (Fig. 6). There was significant heterogeneity between studies, as I^2^ = 83.59%, *p* = 0.00). Longer ICU lengths of stay were witnessed among those treated with antivirals in the Schoninger study [29] and the Cowley study [25].

**Fig. 7:**
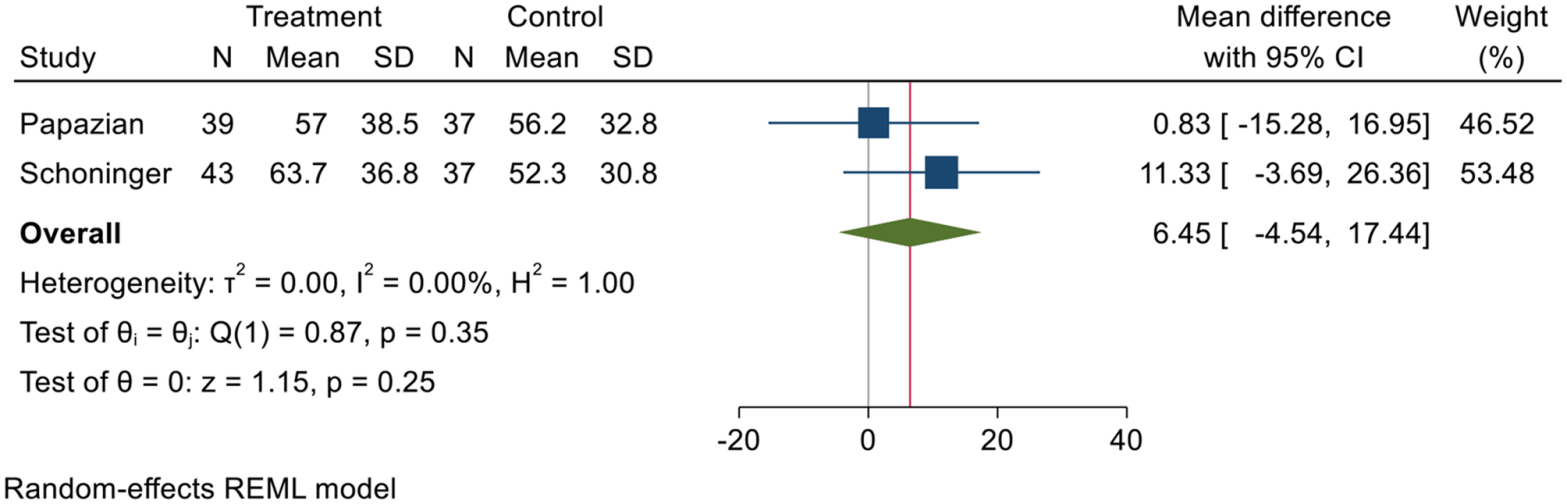
Comparison of mean differences in days of hospitalized duration. SD = standard deviation, CI = confidence interval

The overall duration of hospitalization was comparable between those treated and untreated across studies (mean difference 6.45 days, *p* = 0.25) in the two studies that reported this result (Fig. 7). Heterogeneity was low, with I^2^ value of 0.00%, *p* = 0.35.

The studies used a variety of different outcomes measures to assess whether there is alteration in respiratory support post treatment. Due to marked heterogeneity, no pooled estimate is provided. As can be seen from Table 1, Limaye, Papazian, and Schoninger studies all showed no evidence in duration of mechanical ventilation use between treated and untreated groups, with p values of 0.16, 0.25, and 0.176, respectively. In the Limaye study, the ganciclovir-treated group appeared to have more VFDs by day 28, with a median of 23 (IQR 16–25) compared to 20 (IQR 8–24) in the placebo group; the absolute difference was 3 days (95% CI, 0–6; *p* = 0.05). In contrast, the Papazian study shows no difference in this measure (*p* = 0.256). The Stapleton abstract shows no difference in RSFDs by 28 (*p* = 0.11). Overall, the evidence supporting a reduced need for respiratory support in patients treated with antivirals is weak.

### Quality assessment of the included studies

Overall, the risks of bias in the four randomized controlled studies were assessed as low to moderate and are as listed in Tables 3 and 4. As the Stapleton study is currently only available as an abstract, quality assessment of the study cannot be thoroughly performed. Quality of the two retrospective cohort studies were considered fair, as detailed in Table 5.

**Table 3:**
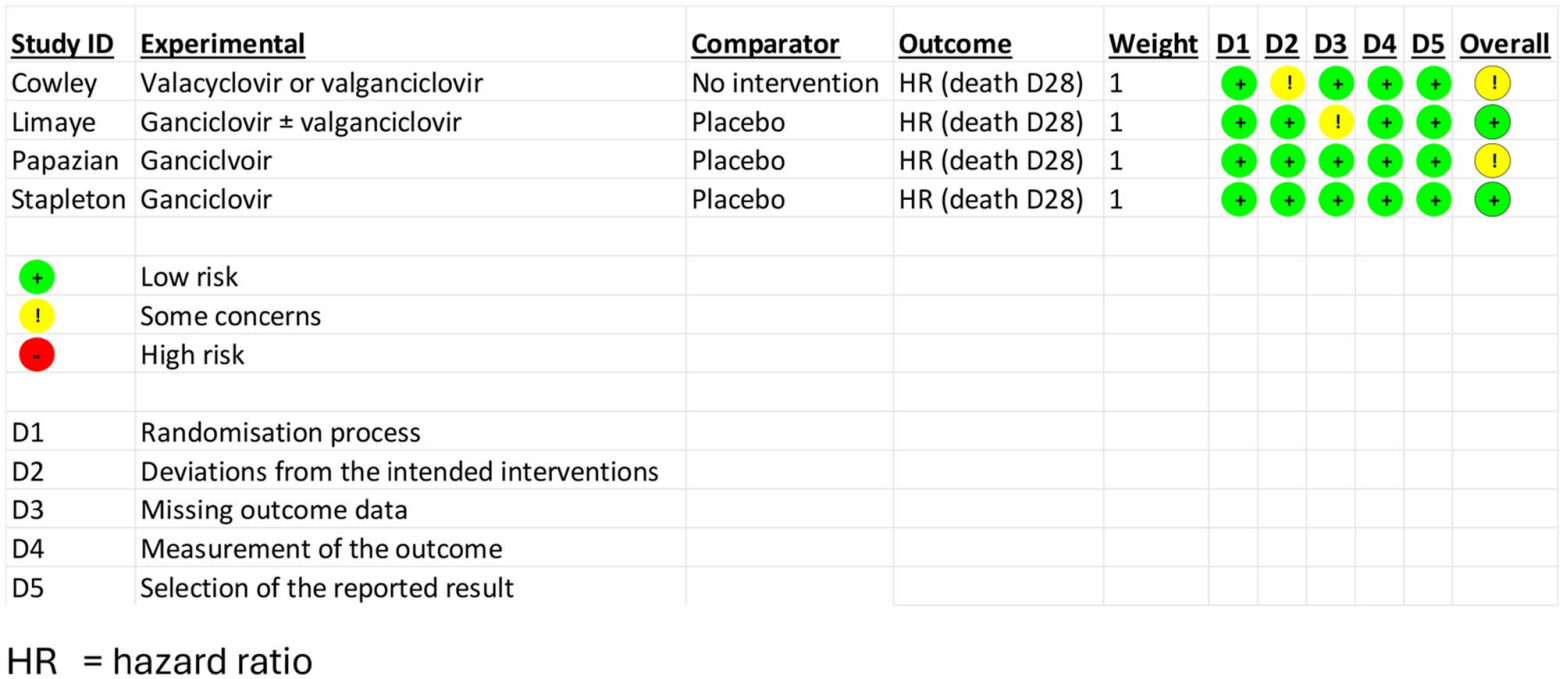
Summary of quality assessment of RCTs, using Cochrane risk of bias Tool, version 2 (RoB 2)

**Table 4:** Quality assessment of the RCTs (using version 2 of Cochrane risk of bias Tool)

	Bias arising from the randomization process	Bias due to deviations from intended interventions	Bias due to missing outcome data	Bias in measurement of the outcome	Bias in selection of the reported result	Overall bias
Cowley 2017 [25]	Computer-generated random treatment allocation sequence Patients and treating physicians not masked to the assigned treatment group, unknown whether they knew their group before being assigned intervention. No significant baseline differences noted in baseline groups.	Patients and treating physicians not masked to the assigned treatment group Carers and people delivering interventions were aware of assigned interventions. Trial was stopped early due to excess mortality, but no switch in groups occurred.	All of the patients randomized were accounted for.	Outcome was measured by physician. Outcome was ascertained from hospital records. Physician was aware of intervention.	Plan for analysis was finalized ahead of data availability. Analysis was performed by intensive care physicians independent of the study team. Study was stopped prematurely because of excess mortality in one arm.	Moderate
Limaye 2017 [26]	Randomization done in a 1:1 ratio via web-based system. Randomization was concealed and stratified by study site and trauma versus severe sepsis. Baseline characteristics of both groups were similar.	Patients were intubated at the time of enrollment and likely blinded. Carers blinded: pharmaceutical company provided the antiviral and matching placebo via central pharmacy.	160 randomized. All 84 in ganciclovir were included in analysis. 72 out of 76 in placebo group included in analysis. 16 lost to follow-up by day 180, 8 lost to follow-up by day 180. Only 156 included in final analysis.	Outcome was assessed by physician Ascertainment of outcome was from hospital records. Outcome assessors likely blinded.	Plan for analysis was finalized ahead of data availability.	Low
Papazian 2021 [27]	Centralized, secure, web-based, randomization system using minimization assigned patients at 1:1 ratio, stratified by study site, mechanical ventilation duration and organ failure score Baseline characteristics at ICU admission and at time of randomization were comparable.	Placebo and ganciclovir were conditioned in similar bottles. Hospital pharmacy or nurse from a different unit distributed the placebo or ganciclovir throughout the study period.	76 were randomized, and 76 included in the primary analysis.	Outcome was assessed by physician Ascertainment of outcome was by hospital record Outcome assessors were blinded.	Data was analyzed according to prespecified plan.	Moderate
Stapleton 2025 [30]	Abstract mentions randomization but no information yet regarding how this was done. Abstract mentions no baseline comparison between two groups.	Study was double-blinded. Method of distribution of study drug was not specified in the abstract. Cumulative incidence of mortality and hazard ratios were calculated.	205 of 213 randomized received at least one dose of the study medication (ganciclovir or placebo)	Outcome was likely assessed by physician. Ascertainment of outcome was likely by hospital record. No full description of whether physicians were blinded was mentioned in the abstract.	Outcome was analyzed per protocol. Outcome was specified as Day 28 mortality. Analysis plan not fully specified. Study was terminated early based on DSMB recommendation due to concerns of futility and increased mortality in the ganciclovir arm.	Moderate

**Table 5:** Quality assessment of the cohort studies using the Newcastle-Ottawa quality assessment form for cohort studies

	Selection				Comparability	Outcome			Overall Quality
Study name (author year)	Representativeness of the exposed cohort	Selection of the non-exposed cohort	Ascertainment of exposure	Demonstration that outcome of interest was not present at start of study	Comparability of cohorts	Assessment of outcome	Follow-up duration	Adequacy of Follow-up	
Park 2021 [28]	Selected group (those died within 30 days were excluded)	Exposed group were sicker, had higher CMV viral load, and had more co-morbidity (longer duration of hemodialysis)	Secure record (one star)	Yes (one star)	The study controls for age, severity scores, and a high CMV viral load (one star)	Outcome was assessed by chart record (one star)	Follow-up was long enough for duration to occur (one star)	Follow-up rate less than 80%	FAIR
Schoninger 2022 [29]	Somewhat representative (one star)	Patients in the treatment group were more likely to be tested for CMV earlier in the ICU stay, more likely to received glucocorticoids and/or tocilizumab, and higher dexamethasone dose, more likely to have a CMV load ≥ 1000 copiesmL, higher levels of AST, ALT, and ferritin.	Secure record (one star)	Yes (one star)	No adjustment was made (only univariate analysis)	Outcome was assessed by chart record (one star)	Follow-up was long enough for duration to occur (one star)	Complete follow up- all subject accounted for (one star); patients still hospitalized at end of study period were excluded	FAIR

## Discussion

This review examines the current evidence regarding the use of antiviral medications against CMV in non-immunocompromised individuals. After a comprehensive search, only six relevant studies were identified: four RCTs and two retrospective cohort studies, highlighting the paucity of research on this topic. Meta-analysis of crude, unadjusted data from these studies showed that antiviral treatment did not improve mortality in non-immunocompromised patients with CMV reactivation. This effect did not differ by study design or antiviral strategy (prophylactic vs. preemptive). Antiviral use also did not affect hospital length of stay or ICU length of stay. No conclusion can be reached regarding whether antiviral use affected amount of respiratory support required by patients. Although a formal test for publication bias could not be performed due to the small number of studies (< 10), the funnel plot did not indicate obvious bias.

Importantly, the Stapleton randomized trial is currently available only as a conference abstract and has not yet undergone full peer review or complete reporting of methods and results. As such, the data from this trial should be interpreted cautiously. Inclusion of abstract-only data in meta-analysis can increase uncertainty because full methodological details and risk-of-bias information could be missing. We therefore emphasize that conclusions based on the pooled analysis that include Stapleton et al. carry additional uncertainty pending full publication of that trial.

To explore the issue of heterogeneity and the issue of uncertainty with the inclusion of the Stapleton trial, we performed a sensitivity analysis excluding the Stapleton abstract (which contributed substantial weight to the pooled mortality estimate). After excluding Stapleton et al., the pooled odds ratio for mortality was 1.20 (95% CI 0.72–2.01; *p* = 0.49) and between-study heterogeneity decreased from I² = 53.47% to I² = 35.34%, suggesting that although Stapleton’s findings influenced the pooled estimate (Supplementary Fig. 1), exclusion of this trial did not alter the overall effect.

### Limitations of the systematic review

This systematic review has several limitations. Only a few studies addressed the topic, including four RCTs. Of the two cohort studies, one (Park) adjusted for confounders, while the other (Schoninger) reported only univariate analyses. Study endpoints varied, necessitating a composite mortality outcome (28-day, 90-day, and in-hospital mortality), although subgroup analysis of RCTs showed no difference in 28-day mortality. Considerable heterogeneity further limits the meta-analysis. Variability existed in patient populations (general ICU, sepsis-associated respiratory failure, COVID-19 pneumonia), definitions of CMV reactivation (cutoff thresholds, sample types), and antiviral strategies (prophylactic, pre-emptive). Even among the four RCTs, the results varied, with one study showing that ganciclovir prophylaxis led to higher mortality [30], the others showing no difference. The presence of substantial heterogeneity even among the four RCTs suggests that differences such as the specific medications used may have influenced the findings. Although random-effects models were applied, the elevated I² values in some analyses indicate that these study-level differences may have affected the pooled estimates. Consequently, the results should be interpreted with caution, as they may not represent a uniform effect of antiviral therapy across all settings. Full publication of the Stapleton study may provide additional detail to help clarify the sources of heterogeneity and enable more accurate interpretation.

### Unanswered questions

The result of this study leaves several questions unanswered.

The first consideration is the implication of antiviral treatment at different levels of viremia. Most studies included in the present meta-analysis enrolled patients with low levels of CMV viremia. The Cowley, Limaye, and Stapleton studies initiated antiviral treatment for all CMV-seropositive patients. In the Cowley study, the median viral load at the first positive PCR ranged from 22 to 95 copies/mL across study arms. Papazian also reported treatment upon detection of any CMV viremia; the assay cut-off in that study was 500 IU/mL. In the Schoninger study, the median maximum CMV viral load was 731 copies/mL (IQR 249–2991), with most patients having low-level viremia (< 1000 copies/mL serum). In the Park study, 48 of 136 patients had CMV PCR > 5000 copies/mL. Only the Park study reported mortality outcomes adjusted for viral load. It remains unclear whether patients with higher levels of viremia might have benefited from treatment. Furthermore, the present meta-analysis cannot provide guidance on the optimal type or dosage of antiviral medication, as the included studies employed different agents and regimens, and the number of studies was insufficient for meaningful subgroup analyses.

## Conclusions and recommendations

Although antiviral therapy reduces CMV reactivation, current evidence does not support routine antiviral therapy for immunocompetent patients with CMV viremia during critical illness and, notably, prophylactic ganciclovir may be associated with potential harm in at least one large trial. Due to limited trials, varied patient groups and antiviral methods, and reliance on an abstract-only study, no firm recommendations can be given; more robust research is needed.

## Appendix A

**Table:** 

Database	Search strings
Medline (searched via Embase engine)	(cmv: ti OR ‘cytomegalovirus’:ti) NOT (‘transplant’:ti OR ‘transplantation’:ti OR ‘hematopoietic’:ti OR ‘solid organ’:ti OR ‘hsct’:ti OR ‘hct’:ti OR ‘hiv’:ti OR ‘lymphoma’:ti OR ‘leukemia’:ti OR ‘pjp’:ti OR ‘pneumocystis’:ti OR ‘malignancy’:ti OR ‘aids’:ti OR ‘lupus’:ti OR ‘colitis’:ti OR ‘child’:ti OR ‘children’:ti OR ‘fetus’:ti OR ‘fetal’:ti OR ‘congenital’:ti OR ‘neonates’:ti OR ‘sle’:ti OR ‘retinitis’:ti OR ‘cirrhosis’:ti OR ‘cancer’:ti OR ‘case’:ti OR ‘review’:ti) AND (‘icu’:ti OR ‘intensive care’:ti OR ‘critical’:ti OR ‘critically’:ti OR ‘preemptive’:ti OR ‘prophylaxis’:ti OR ‘reactivation’:ti OR ‘ganciclovir’:ti OR ‘valganciclovir’:ti OR ‘valaciclovir’:ti OR ‘valacyclovir’:ti OR ‘gancyclovir’:ti OR ‘valgancyclovir’:ti OR ‘letermovir’:ti OR ‘maribavir’:ti OR ‘foscarnet’:ti OR ‘cidofovir’:ti OR ‘antiviral’:ti OR treatment: ti) AND [english]/lim AND ([adult]/lim OR [aged]/lim) AND [medline]/lim
Embase	(cmv: ti OR ‘cytomegalovirus’:ti) NOT (‘transplant’:ti OR ‘transplantation’:ti OR ‘hematopoietic’:ti OR ‘solid organ’:ti OR ‘hsct’:ti OR ‘hct’:ti OR ‘hiv’:ti OR ‘lymphoma’:ti OR ‘leukemia’:ti OR ‘pjp’:ti OR ‘pneumocystis’:ti OR ‘malignancy’:ti OR ‘aids’:ti OR ‘lupus’:ti OR ‘colitis’:ti OR ‘child’:ti OR ‘children’:ti OR ‘fetus’:ti OR ‘fetal’:ti OR ‘congenital’:ti OR ‘neonates’:ti OR ‘sle’:ti OR ‘retinitis’:ti OR ‘cirrhosis’:ti OR ‘cancer’:ti OR ‘case’:ti OR ‘review’:ti) AND (‘icu’:ti OR ‘intensive care’:ti OR ‘critical’:ti OR ‘critically’:ti OR ‘preemptive’:ti OR ‘prophylaxis’:ti OR ‘reactivation’:ti OR ‘ganciclovir’:ti OR ‘valganciclovir’:ti OR ‘valaciclovir’:ti OR ‘valacyclovir’:ti OR ‘gancyclovir’:ti OR ‘valgancyclovir’:ti OR ‘letermovir’:ti OR ‘maribavir’:ti OR ‘foscarnet’:ti OR ‘cidofovir’:ti OR ‘antiviral’:ti OR treatment: ti) AND [english]/lim AND ([adult]/lim OR [aged]/lim) AND [humans]/lim AND [embase]/lim
Scopus	TITLE ( cytomegalovirus OR CMV ) AND ( "ICU" OR "intensive care" OR "critical" OR "critically" OR "preemptive" OR "prophylaxis" OR "reactivation" OR "ganciclovir" OR "valganciclovir" OR "valaciclovir" OR "valacyclovir" OR "gancyclovir" OR "valgancyclovir" OR "letermovir" OR "maribavir" OR "foscarnet" OR "cidofovir" OR "antiviral" OR "treatment" ) AND NOT ( "transplant" OR "transplantation" OR "hematopoietic" OR "solid organ" OR "HSCT" OR "HCT" OR "HIV" OR "lymphoma" OR "leukemia" OR "PJP" OR "Pneumocystis" OR "malignancy" OR "AIDS" OR "lupus" OR "colitis" OR "child" OR "children" OR "fetus" OR "fetal" OR "congenital" OR "neonates" OR "SLE" OR "retinitis" OR "cirrhosis" OR "cancer" OR "case" OR "review" ) AND (LIMIT-TO (LANGUAGE , "English" ))
CINAHL	TI (“Cytomegalovirus” or “CMV) NOT TI (“transplant” or “transplantation” or “hematopoietic” or “solid organ” or “HSCT” or “HCT” or “HIV” or “lymphoma” or “leukemia” or “PJP” or “Pneumocystis” or “malignancy” or “AIDS” or “lupus” or “colitis” or “child” or “children” or “fetus” or “fetal” or “congenital” or “neonates” or “SLE” or “retinitis” or “cirrhosis” or “cancer” or “case” or “review”) AND TI (“ICU” or “intensive care” or “critical” or “critically” or “preemptive” or “prophylaxis” or “reactivation” or “ganciclovir” or “valganciclovir” or “valaciclovir” or “valacyclovir” or “gancyclovir” or “valgancyclovir” or “letermovir” or “maribavir” or “foscarnet” or “cidofovir” or “antiviral” or “treatment”) limited to English all adults
ClinicalTrials.gov	cytomegalovirus | Other terms: CMV | Adult (18–64), Older adult (65+) | Studies with results
ICTRP Search Portal	“CMV” or “cytomegalovirus” limited to With results only

## Supplementary Information

Below is the link to the electronic supplementary material.

Supplementary Material 1

## Data Availability

Availability of data and material: Data is provided within the manuscript.
