# Supplementary material for: Does treatment with antiviral medication affect mortality among immunocompetent individuals with cytomegalovirus reactivation? A systematic review and meta-analysis
Source: BMC Infect Dis. 2025 Nov 27;25:1664. doi: 10.1186/s12879-025-12205-6 (PMC12659282; doi:10.1186/s12879-025-12205-6)
Supplement: Supplementary file 1 — Supplementary Material 1 [file 12879_2025_12205_MOESM1_ESM.docx]

Supplementary Figure 1.

Favors treatment

Favors control

Odds ratio of composite mortality after using antiviral treatment, excluding Stapleton abstract. CI: confidence interval.
